# Endogenous fungal endophthalmitis: risk factors, clinical features, and treatment outcomes in mold and yeast infections

**DOI:** 10.1186/1869-5760-3-60

**Published:** 2013-09-20

**Authors:** Jayanth Sridhar, Harry W Flynn, Ajay E Kuriyan, Darlene Miller, Thomas Albini

**Affiliations:** 1 Department of Ophthalmology, Bascom Palmer Eye Institute, University of Miami Miller School of Medicine, Miami, FL 33136, USA

**Keywords:** Infectious endophthalmitis, Fungal eye infections, Candida, Aspergillus

## Abstract

**Background:**

The purpose of the current study was to analyze risk factors, clinical features, and treatment outcomes in patients with endogenous fungal endophthalmitis with yeast and mold infections. For this retrospective consecutive case series, microbiologic and clinical records were reviewed to identify all patients with intraocular culture-proven endogenous fungal endophthalmitis treated at a single institution between January 1, 1990 and December 31, 2011.

**Results:**

Sixty-seven eyes of 53 patients were identified; 51 eyes of 39 patients had positive cultures for yeast and 16 eyes of 14 patients had positive cultures for molds. Patients with molds as a causative organism had significantly shorter duration of symptoms prior to diagnosis (molds 3.8 days, yeast 21.0 days, *p* = 0.002), were more likely to be receiving iatrogenic immunosuppression (molds 57.1%, yeast 7.7%, *p* = 0.001), have a history of whole-organ transplantation (molds 35.7%, yeast 2.6%, *p* = 0.001), and were more likely to have hypopyon at the time of diagnosis (molds 37.5%, yeast 6.0%, *p* = 0.001). Patients with endogenous endophthalmitis caused by molds had significantly worse visual acuity at the time of diagnosis (logMAR visual acuity molds 1.80, yeast 1.15, *p* = 0.008) and at final visit (logMAR visual acuity molds 1.97, yeast 1.05, *p* = 0.005) compared to those patients with yeast as a causative organism. There was no significant difference in the rate of retinal detachment between the two groups (mold 12.5%, yeast 30.6%, *p* = 0.201). Patients with cultures positive for mold were significantly more likely to undergo enucleation (molds 25.0%, yeast 0%, *p* < 0.001).

**Conclusions:**

Systemic risk factors for patients with endogenous fungal endophthalmitis caused by molds were iatrogenic immunosuppression and a history of whole-organ transplantation. Shorter duration of symptoms before diagnosis and higher rates of hypopyon occurred in mold cases. While endogenous fungal endophthalmitis is generally associated with poor visual acuity outcomes, infection with mold species was associated with worse visual acuity on presentation and on final follow-up than infection with yeast species. Enucleation rates were much higher in mold cases.

## Background

Endogenous fungal endophthalmitis is a serious ocular condition associated with poor visual outcomes [1-3]. Most patients with this entity have predisposing systemic risk factors, although it may occur rarely in healthy immunocompetent individuals [4,5]. In previous reports, the most common organism causing endogenous fungal endophthalmitis was the yeast species *Candida albicans*, followed by molds such as *Aspergillus* species [6-8].

The purpose of the current study was to analyze and report differences in risk factors, clinical presentation, and treatment outcomes between patients with endogenous fungal endophthalmitis caused by yeast and patients with endogenous fungal endophthalmitis caused by molds.

## Methods

The Institutional Review Board approval was obtained from the University of Miami Miller, School of Medicine Sciences Subcommittee for the Protection of Human Subjects. A search of the ocular microbiology department database was performed to identify all patients with positive vitreous tap and vitrectomy specimen cultures for fungal species between January 1, 1990 and December 31, 2011. Medical records were subsequently reviewed to include those patients with a clinical course consistent with endogenous fungal endophthalmitis. The included patients’ medical records were reviewed for presenting clinical features, relevant patient past medical history, and treatment outcomes. Some of the patients included in this study were previously reported without statistical comparison in a series published by Lingappan et al. [3]. Fungal cultures and identification were performed as previously described in that series [9,10].

Fungal culture results were considered positive when there was growth of the same organism on two or more solid media at the inoculation site, or when the organism grew on one culture media and was noted on a stained smear (gram, Giemsa, or Gomori methenamine silver) [11]. Treatment and management decisions were made by the individual treating physicians without a predefined study protocol.

For statistical analysis, the Snellen visual acuity (VA) was converted to logarithm of minimal angle of resolution (logMAR) equivalents and VA of count fingers, hand motion, light perception, and no light perception were assigned logMAR values of 1.85, 2.3, 2.7, and 3.0, respectively, as previously described [12,13]. The logMAR VA is presented as mean ± standard deviation. Presenting and final VA was compared between the yeast and mold group using a Student’s *t* test. The difference in presenting VA and last recorded VA was also compared between the two groups using a Student’s *t* test. A Pearson chi-square test was used to compare presenting symptoms/signs, risk factors, initial treatments, enucleations, and complication between the two groups. A *p* value of < 0.05 was considered statistically significant.

## Results

**Figure 1:**
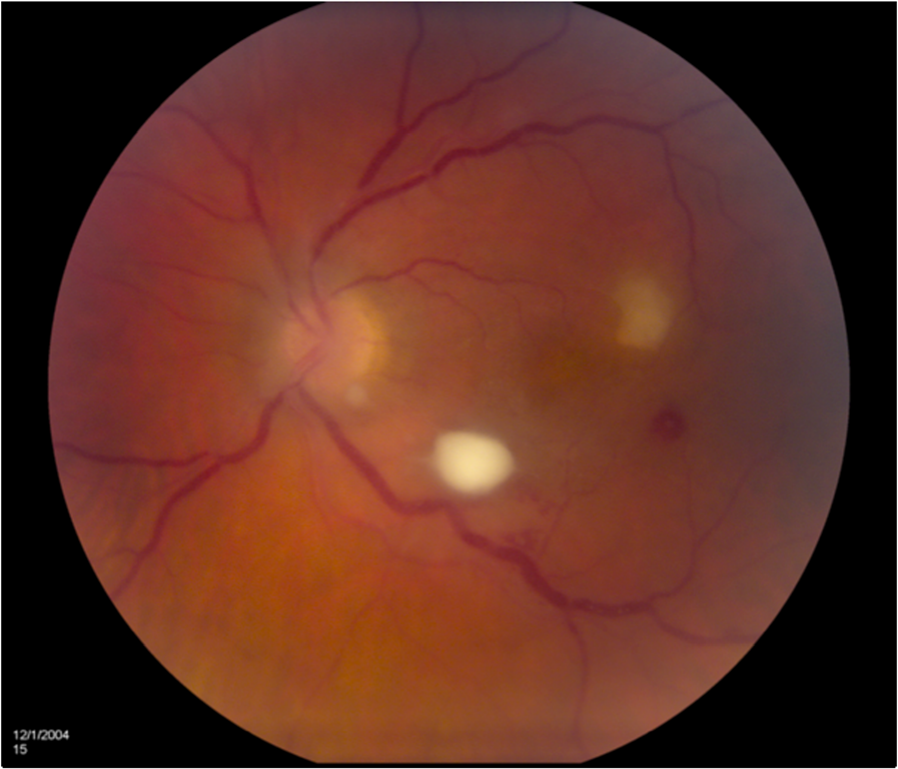
**Endogenous fungal endophthalmitis caused by yeast.** A 34-year-old HIV-positive woman presented with 20/25 vision in the left eye. Fundoscopic examination demonstrated a focal yellow retinal lesion inferotemporal to the optic nerve in the left eye. The patient underwent vitreous paracentesis in the left eye with cultures growing *Candida albicans*. The patient received three intravitreal injections of amphotericin B. Visual acuity on final follow-up was 20/25.

**Figure 2:**
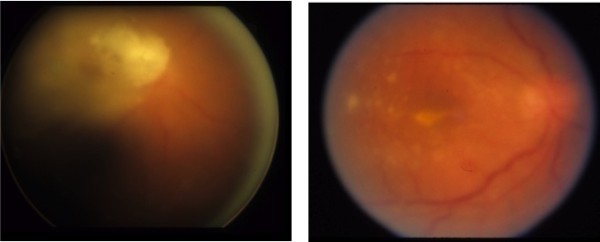
**Endogenous fungal endophthalmitis caused by mold.** A 39-year-old man with history of IV drug use presented with redness and HM vision in the right eye. Fundus examination revealed vitritis and retinitis centered in the macula (above left). The patient underwent pars plana vitrectomy with injection of intravitreal amphotericin B. Pars plana vitrectomy specimen was positive for *Aspergillus glaucus*. Visual acuity on final follow-up was 4/200 (above right).

Over the 22-year study period, 67 eyes of 53 patients were identified as having positive cultures and a clinical diagnosis of endogenous fungal endophthalmitis. Of these, 51 eyes of 39 patients had yeast species and 16 eyes of 14 patients had mold species (Table 1). The mean age of included patients was 50.0 years (range 3 months to 92 years). Mean follow-up was 16.4 months for yeast cases (range 0.25 to 180 months) and 9.6 months (range 0.25 to 34 months). Representative cases are shown in Figures 1 and 2.

**Table 1:** Microbiology of endogenous fungal endophthalmitis

**Organism**	**Cases**
Yeast (39)	
*Candida albicans*	34
*Candida tropicalis*	3
*Cryptococcus neoformans*	2
Mold (14)	
*Aspergillus fumigatus*	7
*Aspergillus glaucus*	2
*Fusarium oxysporum*	2
*Aspergillus terreus*	1
*Aspergillus niger*	1
*Cladophialophora devriessi*	1

A comparison of patient risk factors between the two groups is shown in Table 2. Patients with infection with mold species were significantly more likely to be receiving iatrogenic immunosuppression, including chemotherapy, (molds 57.1%, yeast 7.7%, *p* = 0.001) and have a history of whole-organ transplantation, including cardiac and liver transplantation (molds 35.7%, yeast 2.6%, *p* = 0.001). Positive mold cultures were also significantly associated with having an indwelling venous line or catheter (*p* = 0.010). There was no significant difference between the patient groups in recent hospitalization (*p* = 0.872) or positive systemic cultures (molds 14.3%, yeast 25.6%, *p* = 0.384).

**Table 2:** Systemic risk factors for endogenous fungal endophthalmitis

**Risk factor**	**Yeast (%)**	**Mold (%)**	***p *value**
Recent hospitalization	26/39 (66.7%)	9/14 (64.3%)	0.872
Recent surgery	12/39 (30.8%)	4/14 (28.6%)	0.878
Any immunocompromise	20/39 (51.3%)	9/14 (64.3%)	0.402
Iatrogenic	3/39 (7.7%)	8/14 (57.1%)	*0*.*001*
Diabetes mellitus	7/39 (17.9%)	4/14 (28.6%)	0.589
Cancer	9/39 (23.1%)	3/14 (21.4%)	0.899
HIV	3/39 (7.7%)	0/14 (0.0%)	0.285
Any IV/IVDU/catheter/HD	16/39 (41.0%)	10/14 (71.4%)	*0*.*010*
IVDU	6/39 (15.4%)	4/14 (28.6%)	0.279
Indwelling catheter	3/39 (7.7%)	0/14 (0.0%)	0.285
History of transplant	1/39 (2.6%)	5/14 (35.7%)	*0*.*002*
Positive systemic culture^a^	10/39 (25.6%)	2/14 (14.3%)	0.384

HIV, human immunodeficiency virus; IV, intravenous line; IVDU, intravenous drug use; HD, hemodialysis port; Pearson chi-square test used for statistical analysis. ^a^ Positive systemic cultures for yeast included blood (7), urine (2), and cerebrospinal fluid (1). Positive systemic cultures for mold included sputum (2).

Patients with endogenous endophthalmitis caused by molds were diagnosed significantly closer to onset of symptoms (Table 3, *p* = 0.002). While there was no significant difference between the groups in terms of the type of intraocular inflammation noted by the initial treating physician, patients with positive mold cultures were significantly more likely to have hypopyon at the time of diagnosis (molds 37.5%, yeast 6.0%, *p* = 0.002). Information concerning symptoms and signs was not available for one infant patient.

**Table 3:** Clinical features of endogenous fungal endophthalmitis

**Clinical feature**	**Yeast**	**Mold**	***p *value**
Bilateral	12/39 (30.8%)	2/14 (14.3%)	0.230
Duration of symptoms (mean days ± SD)	21.0 ± 32.9	3.8 ± 5.4	*0*.*002*
Hypopyon	3/50 (6.0%)	6/16 (37.5%)	*0*.*001*
Diffuse anterior and posterior inflammation	33/50 (66.0%)	11/16 (68.8%)	0.839
Anterior inflammation only	2/50 (4.0%)	1/16 (6.3%)	0.707
Posterior inflammation only	15/50 (30.0%)	4/16 (25.0%)	0.701

SD, standard deviation; Pearson chi-square test used for statistical analysis. Note: Information concerning clinical features was not available for one infant patient with endogenous endophthalmitis caused by yeast.

Patients with endogenous endophthalmitis caused by molds were significantly more likely to receive systemic antifungal therapy (Table 4, *p* = 0.035), specifically intravenous antifungal therapy (*p* = 0.011). Systemic intravenous agents included amphotericin B, fluconazole, ketoconazole, and voriconazole. Systemic oral agents included voriconazole and fluconazole. There was no significant difference between the two groups in whether a patient underwent a vitreous tap (*p* = 0.536) or pars plana vitrectomy (molds 50%, yeast 56.9%, *p* = 0.630) as the initial management strategy. Intravitreal antifungal agents used included amphotericin B (5 μg/0.1 ml) and voriconazole (50 μg/0.1 ml). There was no significant difference between the two groups in number of eyes requiring multiple intravitreal injections of antifungals (molds 50%, yeast 33%, *p* = 0.229).

**Table 4:** Initial management strategies for endogenous fungal endophthalmitis

**Initial treatment**	**Yeast (%)**	**Mold (%)**	***p *value**
Local ocular therapy	41/51 (80.4%)	13/16 (81.3%)	0.940
Tap	12/51 (23.5%)	5/16 (31.3%)	0.536
PPV	29/51 (56.9%)	8/16 (50.0%)	0.630
Systemic treatment	30/51 (58.8%)	14/16 (87.5%)	*0*.*035*
Oral	16/51 (31.4%)	4/16 (25.0%)	0.627
Intravenous	14/51 (27.5%)	10/16 (62.5%)	*0*.*011*

Tap: diagnostic vitreous tap; PPV, pars plana vitrectomy with culture; Pearson chi-square test used for statistical analysis.

Treatment outcomes are summarized in Table 5. Patients with endogenous endophthalmitis caused by molds had significantly worse visual acuity on presentation (*p* = 0.008) and on final follow-up (*p* = 0.005). There was no significant difference between the groups in change in visual acuity from presentation to final follow-up (*p* = 0.384) and there was no significant difference in the rate of retinal detachment between the groups (*p* = 0.201). Patients with endogenous endophthalmitis caused by molds underwent enucleation more frequently (molds 25%, yeast 0%, *p* < 0.001). There was no significant difference between mold and yeast subgroups in patient mortality (mold 7.1%, yeast 5.1%, *p* = 0.780).

**Table 5:** Treatment outcomes of endogenous fungal endophthalmitis

**Treatment outcome**	**Yeast**	**Mold**	***p *value**
Visual Acuity (VA)	*n* = 39 (51 eyes)	*n* = 14 (16 eyes)	
Presenting mean	1.15 (± 0.78)	1.80 (± 0.80)	*0*.*008*
logMAR VA (+/− SD)			
Final mean logMAR	1.05 (± 1.01)	1.97 (± 1.18)	*0*.*005*
VA (± SD)			
Change mean	0.11 (± 0.98)	−0.17 (± 1.24)	0.384
logMAR VA (± SD)			
Multiple IVF injections	17/51 (33.3%)	8/16 (50.0%)	0.229
Retinal detachment	15/49 (30.6%)	2/16 (12.5%)	0.201
Cataract	11/49 (22.4%)	1/16 (6.25%)	0.147
Enucleation	0/51 (0.0%)	4/16 (25.0%)	< 0.001

SD, standard deviation; IVF, intravitreal antifungal; Student’s *t* test used for statistical analysis of presenting, final, and change in VA; Pearson chi-square test used for statistical analysis of retinal detachment, cataract formation, and enucleation rate.

## Discussion

The clinical diagnosis of endogenous fungal endophthalmitis has been well described in previous case series and case reports [1,6,14,15]. While there have been descriptions of the differences between patients with yeast infection and patients with mold infection, no large head-to-head statistical comparisons of culture-proven cases have been previously reported [16].

In the current study, endogenous endophthalmitis caused by molds, consisting mainly of *Aspergillus* species, was frequently associated with history of iatrogenic immunosuppression and organ transplantation. In models of invasive aspergillosis impaired neutrophil function has been shown to play a major role in the development of infection, and prolonged steroid use or other immunosuppression may depress neutrophil function [17]. Riddell et al. previously reported in a review of the literature that 43% of patients with endogenous *Aspergillus* endophthalmitis had received prior treatment with corticosteroids [7]. The current study also demonstrated a significantly shorter duration of symptoms for patients presenting with endogenous endophthalmitis caused by molds. Infected eyes were also more likely to have a hypopyon. *Aspergillus* has been shown in histopathologic specimens to prominently invade through blood vessel walls in the choroid leading to necrosis and more rapid involvement of the vitreous [16]. As such, infection with more virulent mold organisms has been shown in the literature to correlate with worse visual acuity outcomes and higher rates of enucleation, as it did in the current study [1,6]. Given the fulminant course of mold infections, patients often require systemic antifungal therapy, as demonstrated in the current study.

In contrast, patients with endogenous endophthalmitis caused by yeast presented more indolently and with better visual acuities, matching descriptions in the literature [18]. Shen et al. previously reported 29 eyes with endogenous fungal endophthalmitis, and none of the mold cases achieving final visual acuity of 20/200 or better; in contrast, 53% of *Candida* cases achieved that outcome [19]. Due to less clear patient histories and nonspecific examination findings mimicking uveitis, misdiagnosis of *Candida* endophthalmitis has been reported to approach 50% [19]. Thus, it is imperative to investigate for risk factors such as recent hospitalization, recent surgery, and intravenous drug use and entertain a diagnosis of fungal endophthalmitis when approaching the uveitis patient with progressive signs and symptoms.

Retinal detachment was a frequent event in the follow-up course for patients in both groups. Retinal detachment is associated with poor visual outcome and is a potential complication of vitrectomy for endophthalmitis [20,21]. One proposed mechanism is post-operative peripheral vitreous contraction inducing retinal breaks [3]. However, in the Endophthalmitis Vitrectomy Study, there was no significant difference in the rate of retinal detachment between patients undergoing needle tap and vitrectomy biopsy [22]. It was recently postulated that early vitrectomy in endogenous endophthalmitis caused by yeast might reduce the incidence of retinal detachment [23]. Sallam et al. reviewed 44 eyes with *Candida* endophthalmitis and reported that eyes that underwent vitrectomy within a week of presentation resulted in a retinal detachment rate of 8% versus 41% in those eyes with delayed vitrectomy.

Pars plana vitrectomy was frequently utilized as the initial management strategy for patients in both groups of the current study. It has been suggested that early vitrectomy is preferable for these patients since vitreous paracentesis may not obtain an adequate vitreous sample of the localized infection. Endogenous fungal endophthalmitis classically begins with choroidal seeding and eventually invades the vitreous cavity [16]. In the current series, the most common isolate was *C*. *albicans* followed by *Aspergillus fumigatus*. This is in accordance with previously reported results [24,25]. Of note, polymerase chain reaction (PCR) testing has shown to be more rapid and sensitive than traditional mycology cultures in diagnosing fungal endophthalmitis and is now employed at many medical centers [26].

In patients with a suspected infection, a diagnostic vitrectomy may be considered initially. Intravitreal therapy can be specifically targeted once appropriate stains and culture results are obtained. Oral antifungal therapy is also considered, usually fluconazole or voriconazole. Depending on the clinical response to initial treatment, intravitreal injections can be given until the infectious process resolves. Patients can be monitored closely for retinal detachment. Although there are early reports proposing the use of intravitreal corticosteroids as an adjunct, there is no well-designed prospective, comparative trial addressing this point and as such, intravitreal steroids are not recommended given the risk of inhibiting the host immune response [27,28].

The current study is limited by its retrospective design as well its relatively small number of patients. Rapid and sensitive PCR testing was not available for clinical use at our institution during the entire study period. Patients had quite variable follow-up and data could be missing from the chart review. Patients were identified based on positive intraocular cultures and thus cases of presumed endogenous endophthalmitis in which cultures were not obtained may have been excluded. In spite of these limitations, this study demonstrates and reinforces key differences between endogenous endophthalmitis caused by molds compared to yeasts.

## Conclusions

Patients with endogenous endophthalmitis caused by molds were more likely to be receiving iatrogenic immunosuppression and have a history of whole-organ transplantation than those patients with endogenous endophthalmitis caused by yeast. Shorter duration of symptoms before diagnosis and higher rates of hypopyon occurred in mold cases. While endogenous fungal endophthalmitis is generally associated with poor visual acuity outcomes, infection with mold species was associated with worse visual acuity on presentation and on final follow-up than infection with yeast species. Enucleation rates were much higher in mold cases.

## Competing interests

HWF is a consultant for Santen and Vindico, and TA is a consultant for Allergan, Bausch and Lomb, Eleven Biotherapeutics, and Thrombogenics. JS, AEK, and DM declare that they have no competing interests.

## Authors’ contributions

JS contributed in the study conception, study design, data collection, and drafting of the manuscript. HWF participated in the study conception, study design, and drafting of the manuscript. AEK contributed to the study design and provided statistical analysis as well as critical revision of the manuscript. DM is the microbiologist for the study and participated in the data collection and critical revision of the manuscript. TA participated in the critical revision of the manuscript. All authors read and approved the final manuscript.
